# Relationship Between Emotional Intelligence and Academic Performance Among Medical Students at University of Tabuk (2021)

**DOI:** 10.7759/cureus.49301

**Published:** 2023-11-23

**Authors:** Omnia S El Seifi, Nada Albishi, Ghadeer A Albalawi, Lena Alzahrani, Lubna I AlOmari, Danah M Albalawi, Shoog M Alharbi, Nouf M Albalawi

**Affiliations:** 1 Family and Community Medicine, University of Tabuk, Tabuk, SAU; 2 Community Medicine, Zagazig University, Zagazig, EGY; 3 Medical School, University of Tabuk, Tabuk, SAU; 4 General Practice, Ministry of Health Saudi Arabia, Tabuk, SAU

**Keywords:** ksa, university of tabuk, medical students, emotional intelligence, academic achievement

## Abstract

Background: There is a strong association between emotional intelligence (EI) and academic performance in medical students.

Objective: This study aimed to explore the ‏ relationship between EI and academic performance among medical students at Tabuk University.

Methods: This cross-sectional study was conducted among medical students in clinical years in the Faculty of Medicine, Tabuk University, Tabuk City, Kingdom of Saudi Arabia (KSA). Each study participant received a self-administered questionnaire composed of two parts: demographic data and the Schutte Self Report Emotional Intelligence Test (SSEIT).

Results: The present study included 203 of the clinical-phase medical students. The academic achievement level was not associated with the students’ gender (p = 0.194) or academic level (p = 0.278). Female students had a significantly higher total SSEIT score than male students (p < 0.001). The sixth-year students had a significantly higher perception of emotion than the fourth-year students (p = 0.029). Students with excellent academic achievement had higher mean scores for managing others’ emotions (p = 0.004) and utilization of emotion compared to those with fair and very good levels (p = 0.042).

Conclusions: Some components of EI correlate with academic performance, gender, and academic level. Further research should be launched to assess the correlation between EI and academic performance among all medical students in all regions of KSA. Students can benefit by attending workshops and courses in universities to develop the students’ EI because of its impact on their academic performance.

## Introduction

Current evidence suggests that emotional intelligence (EI) is associated with enhanced academic performance in different types of professions [1]. Individuals who possess higher EI are more likely to maintain balance and effectively handle challenges in social life [2]. EI is defined as "the ability to monitor one's own and other people's emotions, to discriminate between different emotions and label them appropriately, and to use emotional information to guide thinking and behaviour" [3].

Some of the main competencies for health professionals are closely related to the components of EI [4], emphasizing that EI is a crucial trait for medical students and health professionals [5]. Possessing sound EI enables physicians to communicate more effectively and show empathy during their encounters with patients. This will result in a more interactive and sounder doctor-patient relationship [6], which is a key factor for improving patients’ satisfaction and outcomes.

Research studies described four main components for EI: a) recognition of self-emotions, b) recognition of others’ emotions, c) managing self-emotions, and d) managing others’ emotions [7]. Although some components of EI are inborn talents, improvement can be attained through professional training and can be gained by life experiences [8].

The relationship between academic performance and being a "good" doctor has long been a subject of debate within the medical community. While academic achievements undoubtedly play a crucial role in shaping a doctor's knowledge and skills, there is a lack of concrete evidence linking academic performance directly to the ability to establish and maintain good doctor-patient relationships. In the Kingdom of Saudi Arabia (KSA), some studies were conducted in Riyadh, Taif, and Jeddah [9-11]. However, no similar study was conducted in Tabuk. As EI and the factors affecting its components can vary according to the societies’ characteristics, we conducted the present study at the Faculty of Medicine, Tabuk University, KSA, to explore the‏ relationship between EI and the academic performance of medical students.

## Materials and methods

Ethical considerations

The study obtained approval from the Research Ethics Committee of the Faculty of Medicine, Tabuk University, Tabuk, Saudi Arabia (approval no. UT-269-116-2023).

Participants were informed about the study objectives, methodology, and benefits, and they were informed that participation is completely voluntary. Subjects who agreed to fill the questionnaire implied that they agreed to participate in the study.

The study conserved participants’ privacy. Investigators were responsible for keeping the security of the data. Personal data (e.g., name and contact info) were not entered in our data entry software to conserve the participants' privacy. Each subject got a unique identifier code.

Study design, date, and settings

This cross-sectional survey study was conducted among male and female medical students at the Faculty of Medicine, Tabuk University, Saudi Arabia, during the academic year 2021/2022. Tabuk City is the capital city of the Tabuk Region in Northwestern Saudi Arabia, with a total population of about 657,000 (2020 census). Tabuk campus has separate branches for female and male students, with 800 medical students in total.

Participants' eligibility criteria

As senior students have more experience with academic life, female and male medical students in clinical years (4th-6th) were our participants in this study. Basic-year students and those who refused to participate were excluded from the study.

Sample size and sampling technique 

The sample size was calculated using OpenEpi (https://www.openepi.com/Menu/OE_Menu.htm) based on the total number of students in clinical years (380 students), a 65% prevalence of emotional intelligence from a previous similar study [2], and a 95% confidence limit. The required sample size was 183 medical students at Tabuk University. The sample was obtained by a probability simple random technique from the students’ lists.

Data collection

All medical students received a self-administered questionnaire (see Appendix) to be filled by them with the guidance of data collectors’ notes and instructions at the beginning of the questionnaire. The questionnaire consisted of two parts. The first part included the students' demographics and general characteristic data (age, gender, and GPA). The second part included the Schutte Self-Report Emotional Intelligence Test (SSEIT). The SSEIT is a 33‑item questionnaire that uses a five‑point Likert‑type scale to measure EI traits. The instrument measures the expression of emotion (13 items), regulation of emotion (10 items), and utilization of emotion (10 items), with total scores ranging from 33 to 165 [4].

Statistical analysis

Microsoft Excel program (Microsoft, USA) was used for data entry. Statistical analysis was performed using the Statistical Package for Social Sciences (IBM SPSS Statistics) for Windows, version 26 (IBM Corp., Armonk, N.Y., USA). Categorical variables (e.g., gender and academic level) were presented as counts and percentages. The association between categorical variables was tested using Pearson’s chi-square test for independence. Numerical variables (the EI scores) were summarized as mean ± standard deviation (SD), and the associations between the variables were tested using the independent samples T-test (for two groups) or the one-way analysis of variance test (ANOVA, for three or more groups). A significant p-value from the one-way ANOVA test was followed by a post-hoc Tukey test to assess pairwise comparisons between the groups. The level of significance was set at a p-value <0.05.

## Results

The present study included 203 medical students. Male and female students accounted for 30.5% and 69.5%, respectively. The fourth-year students represented nearly half the sample (49.7%), while the fifth- and sixth-year students accounted for 30.5% and 19.7%, respectively. The GPA of the students was fair in 30.5%, good in 30%, very good in 22.7%, and excellent in 16.7% of the total students.

**Figure 1:**
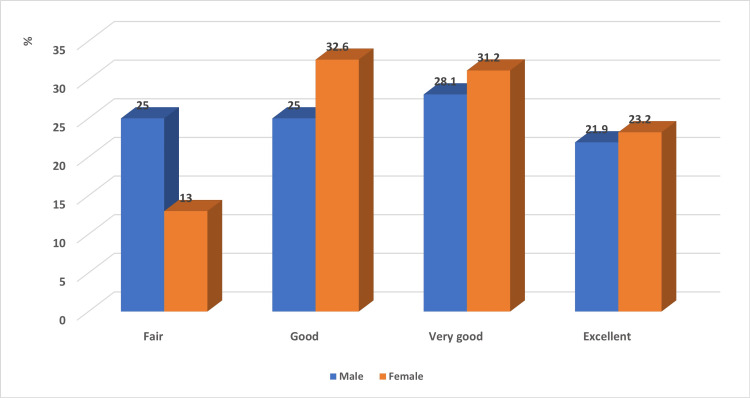
Association of the academic achievement level of medical students with gender Chi square was computed. P value = 0.194.

**Figure 2:**
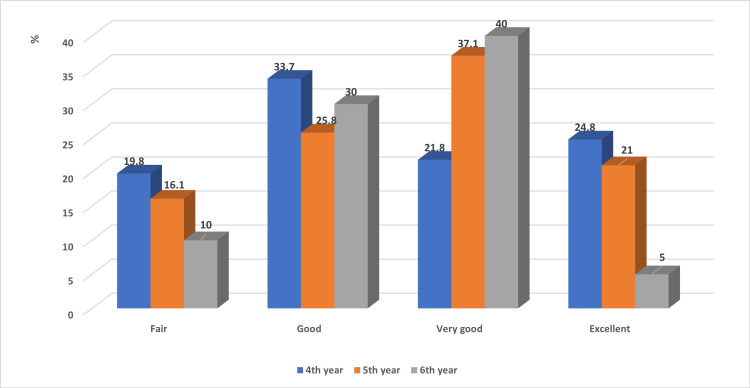
Association of the academic achievement levels of medical students with their academic level Chi square was computed. P value = 0.278.

The medical achievement level of the female students tended to be slightly higher than that of the male students; however, the difference did not reach statistical significance (p = 0.194; Figure 1). Moreover, no statistically significant association was detected between the academic achievement level of the enrolled medical students and their academic level (p = 0.278; Figure 2).

The statistical analysis explored the differences in the total EI score and its subsets in relation to relevant characteristics of the students, including gender, academic level, and academic achievement level.

The female students had a significantly higher total EI score compared to the male students (83.93 ± 9.3 vs. 78.23 ± 10.6, respectively; p < 0.001). A comparison of the subsets of the scores revealed that the perception of emotion and managing own emotions did not significantly differ according to gender (p = 0.234 and 0.099, respectively). Meanwhile, the female students showed a significantly higher mean values of managing others’ emotions (30.55 ± 4.3 vs. 27.76 ± 4.3; p < 0.001) and utilization of emotion (15.66 ± 2.4 vs. 14.06 ± 2.5; p < 0.001 (Table 1)).

**Table 1:** Comparison of the mean score of emotional intelligence among male and female medical students at the University of Tabuk T-test was computed. *P< 0.05 is significant.

Emotional intelligence domains/gender	Female	Male	P
Perception of emotion	13.65 ± 2.1	13.25 ± 2.3	0.234
Managing own emotions	24.05 ± 3.6	23.15 ± 3.4	0.099
Managing others’ emotions	30.55 ± 4.3	27.76 ± 4.3	0.000*
Utilization of emotion	15.66 ± 2.4	14.06 ± 2.5	0.000*
Total emotional intelligence	83.93 ± 9.3	78.23 ± 10.6	0.000*

As regard the relationship of EI with the students’ academic levels, no significant differences in the total score were observed (p = 0.223), despite the slightly higher mean value of the sixth-year students. Likewise, there was no significant differences in the subscales of managing own emotions (p = 0.092), managing others’ emotions (p = 0.645), and utilization of emotions (p = 0.164). However, sixth-year students showed a significantly higher perception of emotion than fourth-year students (14.32 ± 2.0 vs. 13.25 ± 2.3; p = 0.029 (Table 2)).

**Table 2:** Comparison of the mean score of emotional intelligence among the medical students according to their academic level ANOVA test was computed. *P<0.05 is significant. a: significant difference from the fair group on the post-hoc test; b: significant difference from the very good group on the post-hoc test.

Emotional intelligence	4^th^ year (N = 101)	5^th^ year (N = 62)	6^th^ year (N = 40)	P
Perception of emotion	13.25 ± 2.3 ^b^	13.43 ± 2.0	14.32 ± 2.0 ^a^	0.029*
Managing own emotions	23.73 ± 3.6	23.17 ± 3.3	24.75 ± 3.8	0.092
Managing others’ emotions	29.51 ± 5.0	29.56 ± 3.8	30.27 ± 4.1	0.645
Utilization of emotion	14.85 ± 2.6	15.61 ± 2.3	15.20 ± 2.4	0.164
Total emotional intelligence	81.35 ± 10.7	81.79 ± 9.0	84.55 ± 9.7	0.223

The various academic achievement levels exhibited no difference in the total EI score (p = 0.411), nor in the perception of emotion (p = 0.755) and managing own emotions (p = 0.622). Meanwhile, students with excellent academic achievement had higher mean scores for managing others’ emotions (p = 0.004) and utilization of emotions compared to those with fair and very good levels (p = 0.042 (Table 3)).

**Table 3:** Comparison of the mean score of emotional intelligence among the levels of academic achievement ANOVA test was computed. *P<0.05 is significant. a: significant difference from the fair group on the post-hoc test; b: significant difference from the very good group on the post-hoc test; c: significant difference from the excellent group on the post-hoc test

Emotional intelligence	Fair (N = 34)	Good (N = 62)	Very good (N = 61)	Excellent (N = 46)	P
Perception of emotion	13.41 ± 2.1	13.64 ± 2.0	13.31 ± 2.5	13.71 ± 2.0	0.755
Managing own emotions	23.50 ± 3.1	23.41 ± 3.7	23.88 ± 3.8	24.26 ± 3.3	0.622
Managing others’ emotions	28.91 ± 4.5 ^c^	29.80 ± 4.5	29.04 ± 4.3 ^c^	31.91 ± 4.5 ^a,b^	0.004*
Utilization of emotion	14.94 ± 2.9 ^c^	15.24 ± 2.1	15.09 ± 2.5 ^c^	16.26 ± 2.3 ^a,b^	0.042*
Total emotional intelligence	80.76 ± 10.9	82.11 ± 9.6	81.34 ± 10.3	84.15 ± 9.5	0.411

## Discussion

Previous studies commonly reported a strong correlation between EI and academic performance in medical students [6,10- 13]. The current study aimed to find out the relationship between EI and academic performance among male and female medical students at the Faculty of Medicine, Tabuk University, Tabuk, KSA. Several factors might affect EI, including gender, age, and academic level [13]. Consequently, the current study investigated the effect of these variables on EI.

We found that EI differs by gender as the female students had a significantly higher total EI score compared to the male students (83.93 ± 9.3 vs. 78.23 ± 10.6; p < 0.001). These significant differences were attributed to the components of managing others’ emotions (30.55 ± 4.3 vs. 27.76 ± 4.3; p < 0.001) and utilization of emotion (15.66 ± 2.4 vs. 14.06 ± 2.5; p < 0.001).

This finding agrees with other studies conducted in KSA [10] and other countries [14-16], which reported higher mean scores of EI in female students [14,10,16,17]. A recent systematic review [13] reported that based on six out of seven studies, female medical students have higher EI. Meanwhile, studies showed variations in the reported subscales and their significance. Austin et al. [14] in the United Kingdom and Ibrahim et al. [10] in Saudi Arabia found that females obtained significantly better mean scores in all EI domains compared to males, while Wijekoon et al. [17] in Sri Lanka, similar to our results, reported significant differences in some subscales only.

The higher EI exhibited by female students may be attributed to the more emotional nature of females, and they are used to sharing emotional conversations with their families more than males do [18]. Meanwhile, studies in the KSA by Alghamdi [19] in Albaha, Naeem et al. [9] at three medical colleges in KSA, and Altwijri et al. [11] in Riyadh showed a lack of gender effect of EI. Likewise, studies from Pakistan [20] and South India [21] revealed no significant differences in EI scores between male and female students. A recent study from Iran [22] reported that male students had a significantly higher mean total EI score than females. These contradictory findings may be attributed to differences in the societal and cultural factors in the populations from which the study samples were drawn. In some societies, females have restricted interaction with people outside the family and school circles, thereby limiting their emotional experiences with others [22]. Another possible explanation is the difference in time when the studies were conducted as societies show changes in socialization, and new cultural concepts are introduced by the lapse of time.

The current study results demonstrated a lack of significant differences in total EI scores among the students’ academic levels (p = 0.223). The sixth-year students exhibited a slightly higher mean total score, but they showed a significantly higher perception of emotion than the fourth-year students (14.32 ± 2.0 vs. 13.25 ± 2.3; p = 0.029). This could be explained by gaining EI skills by learning or through life experience [8]. It seems that some subscales of EI undergo development and improvements while other subscales may either show delay or non-improvement until specific training is performed. In partial agreement with these results, Naeem et al. [9], Ranasinghe et al. [16], and Altwijri et al. [11] reported lack of significant differences among the clinical years' students regarding the overall EI score or its subscales. The systematic review by Karkada et al. [13] reported no difference in EI among the academic levels. However, Ibrahim et al. [10] revealed that students in higher educational years had significantly better EI scores compared to others, but they compared second-year students to those in the third year and up to the sixth year. 

The current study found that the total EI score did not vary significantly by the level of academic achievement (p = 0.411). However, students with excellent academic achievement had higher mean scores for managing others’ emotions (p = 0.004) and utilization of emotions compared to those with fair and very good levels (p = 0.042). The lack of association between academic achievement level and total EI coincides with the findings of earlier studies [16,19].

Meanwhile, other studies reported a positive correlation between the total EI and academic achievement levels [6,10,11,22]. Naeem et al. [9] found a significant positive correlation only between cumulative GPA and the EI of medical students (r = 0.246; p < 0.000) on the optimism subscale, while no correlation was observed between cumulative GPA and awareness of emotions or use of emotion subscales. A recent systematic review by Singh et al. [23] found, based on the findings of nine studies, a significant relationship between EI and GPA in clinical years. However, EI did not correlate with the scores of academic performances in the preclinical years.

The effect of EI on academic performance may be due to better communication, teamwork, and time management skills that are required in several educational activities [24,25]. Higher EI is also associated with better critical thinking [26] and enhanced adaptability and proactivity during their encounters with patients and residents [27]. These traits and skills are essential for medical students, particularly with the increased dependence on self-directed learning and student-centred learning strategies. Moreover, EI is associated with emotional well‑being, leading to better stress management, which in turn may improve the students’ academic performance [6,28].

In the current study, analysis of the results showed that the emotional entillegence was not associated with students’ gender (p = 0.194) or academic level (p = 0.278). Therefore, a multivariate analysis was not conducted to adjust for their effects on academic performance.

The present study bears several points of strength. It is the first study of this type conducted at Tabuk University. In addition, the study explored the relationship of relevant variables (gender and academic level) with academic achievement and EI. Nevertheless, the study possessed certain limitations. All students participating in the study belonged to a single institution, which may have limited the generalization of the results. The GPA was self‑reported, so its accuracy cannot be guaranteed, and this may introduce bias.

## Conclusions

This study showed that there is a correlation between EI and the academic performance of the medical students at Tabuk University. EI was higher in the female than male medical students. We recommend the conduction of further research to evaluate EI intervention studies through the incorporation of EI within the medical curriculum. In addition, conducting workshops and courses is recommended to develop students’ EI because of its impact on their academic performance.

## Appendices

 Questionnaire (Arabic version)

１-  هل أنت ؟ ذكر / أنثى

２-  ما هي سنتك الدراسية الحالية ؟ ( السنة الرابعة / السنة الخامسة / السنة السادسة )

３-  ما هو معدلك الجامعي التراكمي (GPA) من ٥ ؟ ( من ٤.٥٠ الى ٥ / من ٤ الى ٤.٤٩ / من ٣.٥٠ الى ٤ / من ٣ الى ٣.٤٩ )

SSEIT (Schutte Self-Report Emotional Intelligence Test)

عزيزي الطالبـ/ـة لا يوجد هناك إجابة صحيحة او خاطئة ، رجاءًا اجب على اكثر خيار يصفك .

١: غير موافق ابدًا

٢: غير موافق لحد ما

٣: محايد

٤: موافق لحد ما

٥: موافق جدًا

**Table 4:** السؤال

السؤال	١	٢	٣	٤	٥
أعلم متى أتحدث عن مشاكلي الشخصية للآخرين.					
عندما أواجه عقبات ، أتذكر الأوقات التي واجهت فيها عقبات مماثلة وتغلبت عليها.					
أتوقع أنني سأبلي بلاءً حسناً في معظم الأشياء التي أجربها.					
يجد الآخرون أنه من السهل أن يثقوا بي.					
دفعتني بعض الأحداث الكبرى في حياتي إلى إعادة تقييم ما هو مهم وغير مهم.					
عندما يتغير مزاجي ، أرى إمكانيات/ احتمالات جديدة.					
العواطف هي أحد الأشياء التي تجعل حياتي تستحق العيش.					
أنا مدرك لمشاعري ، كما أني أعيشها.					
أحب مشاركة مشاعري مع الآخرين.					
عندما أشعر بمشاعر إيجابية ، أعرف كيف أجعلها تدوم.					
أنظم المناسبات التي يستمتع بها الاخرون.					
أبحث عن الأنشطة التي تجعلني سعيدًا.					
أقدم نفسي بطريقة تترك انطباعًا جيدًا لدى الآخرين.					
انا اعرف مشاعر الأخرين ين بالنظر اليهم فقط.					
أعرف لماذا تتغير مشاعري.					
عندما أكون في مزاج إيجابي , أستطيع ابتكار أفكار جديدة.					
أنا أتحكم في مشاعري.					
أثني على الأخرين عندما يقوموا بعمل جيد.					
عندما يخبرني شخص آخر عن حدث مهم في حياته , أشعر وكأنني عايشت هذا الحدث بنفسي.					
عندما يواجهني تحدي، استسلم لأنني مؤمن بأنني سأفشل.					
انا اساعد الآخرين على الشعور بالتحسن عندما يشعرون بالاحباط.					
أستخدم المزاج الجيد لمساعدة نفسي على الاستمرار في المحاولة في مواجهة العقبات.					
من الصعب بالنسبة لي أن أفهم لماذا يشعر الناس بالطريقة التي يشعرون بها.					

ALSAQ

عزيزي الطالبـ/ـة لا يوجد هناك إجابة صحيحة او خاطئة ، رجاءًا اجب على اكثر خيار يصفك .

١: غير موافق ابدًا

٢: غير موافق لحد ما

٣: محايد

٤: موافق لحد ما

٥: موافق جدًا

**Table 5:** السؤال

السؤال	١	٢	٣	٤	٥
أفعالي تعكس قيمي الأساسية.					
أسعى للحصول على آراء الآخرين قبل اتخاذ قراري الخاص.					
لا اسمح بضغط المجموعة ان يتحكم بي.					
أستمع جيدا لأفكار من يختلفون معي.					
أسعى للحصول على آراء الآخرين كطريقة لفهم نفسي .					
نادرا ما أظهر نفسي على خطأ أمام الآخرين .					
أتقبل شعوري تجاه نفسي.					
أخلاقي توجه ما أفعله كقائد.					
أعترف بأخطائي للآخرين .					

Questionnaire (English version)

1. Are you (male/female)?

2. What is your current academic year? (4th year, 5th year, 6th year)

3. What is your GPA out of 5? (4.5-5, 4-4.49, 3.5-4, 3-3.49)

SSEIT

Dear student, there are no right or wrong answers. Please give the response that best describes you.

 1 = strongly disagree

2 = somewhat disagree

3 = neither agree nor disagree

4 = somewhat agree

5 = strongly agree

**Table 6:** SSEIT (Schutte Self-Report Emotional Intelligence Test)

5	4	3	2	1	
					I know when to speak about my personal problems to others.
					When I am faced with obstacles, I remember times I faced similar obstacles and overcame them.
					I expect that I will do well on most things I try.
					Other people find it easy to confide in me
					Some of the major events of my life have led me to re-evaluate what is important and not important
					When my mood changes, I see new possibilities.
					Emotions are one of the things that make my life worth living.
					I am aware of my emotions as I experience them.
					I like to share my emotions with others.
					When I experience a positive emotion, I know how to make it last.
					I arrange events others enjoy.
					I seek out activities that make me happy.
					I present myself in a way that makes a good impression on others.
					By looking at their facial expressions, I recognize the emotions people are experiencing.
					I know why my emotions change.
					When I am in a positive mood, I am able to come up with new ideas.
					I have control over my emotions.
					I compliment others when they have done something well.
					When another person tells me about an important event in his or her life, I almost feel as though I experienced this event myself.
					When I am faced with a challenge, I give up because I believe I will fail.
					I help other people feel better when they are down.
					I use good moods to help myself keep trying in the face of obstacles.
					It is difficult for me to understand why people feel the way they do.

ALSAQ

Dear student, there are no right or wrong answers. Please give the response that best describes you.

1=Strongly disagree

2=Disagree

3=Neutral

4=Agree

5=Strongly agree 

**Table 7:** ALSAQ

5	4	3	2	1	
					My actions reflect my core values
					I seek others’ opinions before making up my own mind
					I do not allow group pressure to control me
					I listen closely to the ideas of those who disagree with me
					I seek feedback as a way of understanding who I really am as a person
					I rarely present a “false” front to others
					I accept the feelings I have about myself
					My morals guide what I do as a leader
					I admit my mistakes to others
